# Increased Indoleamine 2,3-Dioxygenase Levels at the Onset of Sjögren’s Syndrome in SATB1-Conditional Knockout Mice

**DOI:** 10.3390/ijms221810125

**Published:** 2021-09-19

**Authors:** Yuriko Tanaka, Mayu Onozato, Tetuo Mikami, Terumi Kohwi-Shigematsu, Takeshi Fukushima, Motonari Kondo

**Affiliations:** 1 Department of Molecular Immunology, Toho University School of Medicine, Tokyo 143-8540, Japan; yurikota@med.toho-u.ac.jp; 2 Department of Analytical Chemistry, Faculty of Pharmaceutical Sciences, Toho University, Chiba 274-8510, Japan; mayu.onozato@phar.toho-u.ac.jp (M.O.); t-fukushima@phar.toho-u.ac.jp (T.F.); 3 Department of Pathology, Toho University School of Medicine, Tokyo 143-8540, Japan; tetsuo.mikami@med.toho-u.ac.jp; 4 Department of Orofacial Sciences, University of California San Francisco School of Dentistry, San Francisco, CA 94143, USA; terumi.kohwi-shigematsu@ucsf.edu

**Keywords:** autoimmunity, Sjögren’s syndrome, interferon γ, tryptophan metabolism

## Abstract

Sjögren’s syndrome (SS) is a chronic autoimmune disease characterized by dysfunction of salivary and lacrimal glands, resulting in xerostomia (dry mouth) and keratoconjunctivitis sicca (dry eyes). Autoantibodies, such as anti-SSA and anti-SSB antibodies, are hallmarks and important diagnostic factors for SS. In our previous study, we demonstrated that SS-like xerostomia was observed in SATB1 conditional knockout (SATB1cKO) mice, in which the floxed SATB1 gene was specifically deleted in hematopoietic cells as early as 4 weeks of age. In these mice, autoantibodies were not detected until 8 weeks of age in SATB1cKO mice, although exocrine gland function reached its lowest at this age. Therefore, other markers may be necessary for the diagnosis of SS in the early phase. Here, we found that mRNA expression of the *interferon* *γ* (*IFN-γ*) gene and the IFN-responsive *indoleamine 2,3-dioxygenase* (*IDO*) gene is upregulated in the salivary glands of SATB1cKO mice after 3 and 4 weeks of age, respectively. We detected l-kynurenine (l-KYN), an intermediate of l-tryptophan (l-Trp) metabolism mediated by IDO, in the serum of SATB1cKO mice after 4 weeks of age. In addition, the upregulation of IDO expression was significantly suppressed by the administration of IFN-γ neutralizing antibodies in SATB1cKO mice. These results suggest that the induction of IFN-dependent IDO expression is an initial event that occurs immediately after the onset of SS in SATB1cKO mice. These results also imply that serum l-KYN could be used as a marker for SS diagnosis in the early phases of the disease before autoantibodies are detectable.

## 1. Introduction

Sjögren’s syndrome (SS) is a systemic autoimmune disease in which the salivary and lacrimal glands are functionally impaired [1]. As a result, SS patients suffer from xerostomia (dry mouth) and keratoconjunctivitis sicca (dry eye) [2]. Since any damage to the exocrine glands is functionally and structurally irreversible, SS patients require moisturizing treatments of the mouth and eyes throughout their lives [3]. Therefore, early diagnosis of SS is important for appropriate treatment of the disease to ensure a better quality of life.

Special AT-rich sequence binding protein-1 (SATB1) is a chromatin organizer that positively and negatively regulates the expression of various genes [4,5]. SATB1 is expressed by the earliest hematopoietic stem cells (HSCs) and is necessary to maintain HSC stemness [6,7]. Although SATB1 is expressed in various hematopoietic cell types, such as dendritic cells [8,9], SATB1 expression in T-lineage cells has garnered interest. This is because SATB1 expression is higher in T lineage cells than in other types of hematopoietic cells [9,10], especially CD4/CD8 double-positive thymocytes, in which positive selection events occur [11,12]. Indeed, positive selection is impaired in both SATB1 null and hematopoietic cell-specific SATB1 conditional knockout (SATB1cKO) mice, which were used in this study [10,11]. In addition, negative selection events are impaired in the absence of SATB1 [11]. Furthermore, the development of regulatory T (Treg) cells in the thymus is blocked in SATB1cKO mice [11,13,14], suggesting that SATB1 is necessary to establish immune tolerance.

**Figure 1:**
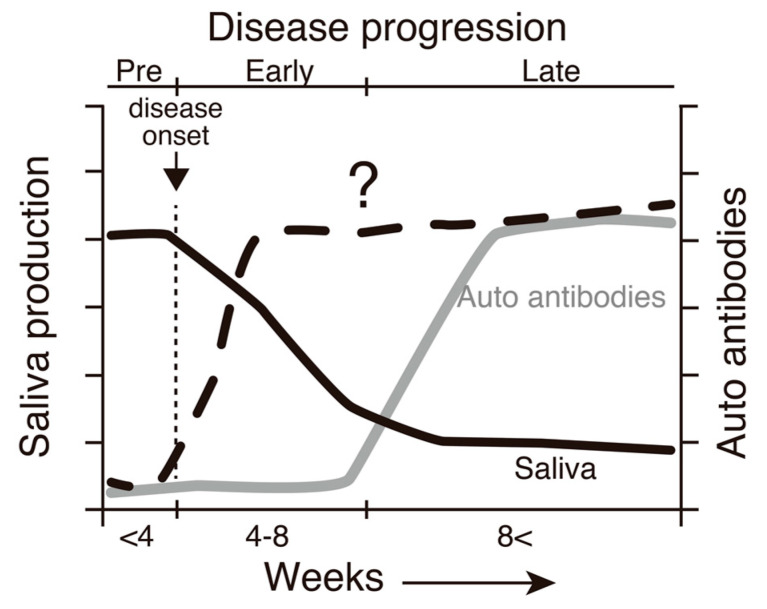
A schematic diagram demonstrating a correlation between SS-like pathogenesis and the age of SATB1cKO mice based on our previous investigation [16]. A decrease in saliva production is initiated at 4 weeks of age (solid line). The production levels reach the basal level at ~8 weeks of age, when autoantibodies, such as anti-SSA and anti-SSB antibodies, are detected in the serum (dotted line). Therefore, we can distinguish the three distinct SS stages in SATB1cKO mice: pre (before 4 weeks of age), early (between 4 and 8 weeks old) and late (older than 8 weeks after birth). In the present study, we examined factors whose levels may be elevated in the early phase of the disease in SATB1cKO mice (gray line).

Disturbances in immune tolerance may lead to autoimmune reactions [15]. In a previous study, SATB1cKO mice begin to die after 24 weeks of age due to renal failure, most likely caused by lupus nephritis associated with systemic lupus erythematosus (SLE) [11]. Notably, salivary gland dysfunction is observed in SATB1cKO mice as early as 4 weeks of age, with the maximum severity observed at ~8 weeks after birth [16]. Immune cell infiltration and the destruction of the salivary gland structure were observed in SATB1cKO mice, which is also a characteristic feature observed in patients with SS [16]. Therefore, SATB1cKO mice were used as the mouse model for SS. Since individual variations in the onset and progression of SS disease have been observed in other SS model mice, SATB1cKO mice are useful tools for analyzing SS pathogenesis, especially in the early phase of the disease. We previously demonstrated that the levels of anti-SSA and anti-SSB antibodies, which are used in the clinical diagnosis of SS, are upregulated when saliva production reaches its lowest levels in SATB1cKO mice [16]. This finding implies that markers other than the presence of autoantibodies are necessary to properly diagnose SS in the early phase of the disease when the exocrine function is maintained (see Figure 1).

Here, we found that the levels of l-kynurenine (l-KYN), an intermediate in the breakdown of l-tryptophan (l-Trp) [17], were upregulated in the serum of SATB1cKO mice as early as 4 weeks of age. The expression of indoleamine 2,3-dioxygenase (IDO, encoded by the *IDO1* gene) [18], which mediates the generation of l-KYN from l-Trp, was upregulated in the salivary glands of 4-week-old SATB1cKO mice, suggesting that the induction of IDO expression might be one of the earliest responses in exocrine glands injured by an autoimmune reaction. The presence of l-KYN in serum could be a noninvasive diagnostic marker for SS before the deterioration of the exocrine gland function.

## 2. Results

### 2.1. Expression of IFN-γ Is Upregulated in the Salivary Gland of SATB1cKO Mice upon Reduction in Saliva Production 

**Figure 2:**
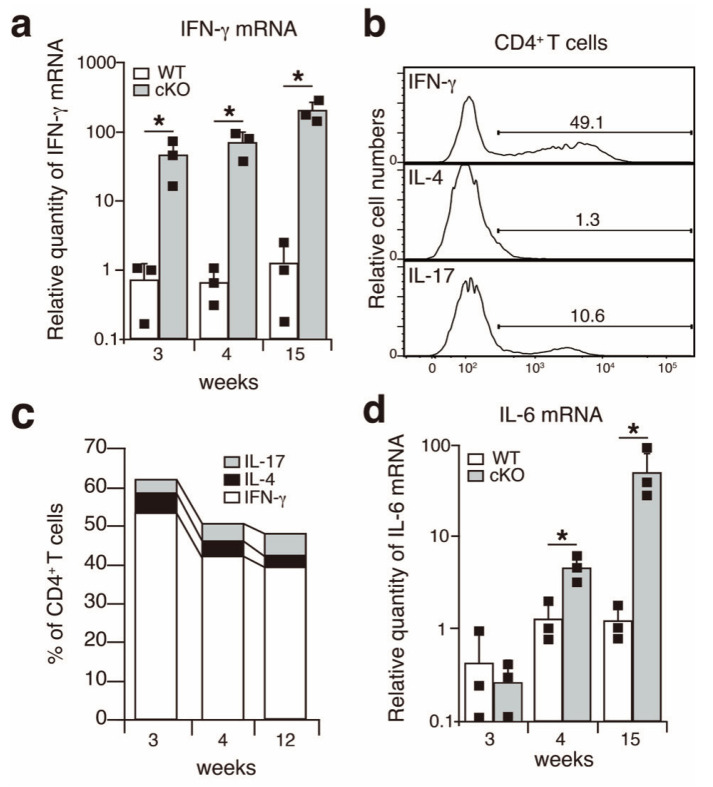
IFN-γ is upregulated in the salivary glands of SATB1cKO mice. (**a**,**d**) Quantitative PCR analyses of the expression of IFN-γ (**a**) and IL-6 (**d**) in the salivary glands of WT (open bar) and SATB1cKO mice (filled bar). Each result was normalized to the expression level of HPRT. Dots in the graph indicate individual values (*n* = 3). The Student’s *t*-test was used for statistical analysis (* *p* < 0.05). (**b**) A representative FACS plot showing the expression of IFN-γ, IL-4 and IL-17 upon infiltration of CD4^+^ T cells in the salivary glands of SATB1cKO mice in the late SS phase (12 weeks old). (**c**) The percentage of CD4^+^ T cells expressing IFN-γ (open), IL-4 (filled with black) and IL-17 (filled with grey) that infiltrated the salivary gland of SATB1cKO mice at 3, 4 and 12 weeks after birth. The mean value (*n* = 3–5) is shown.

We previously demonstrated that SATB1cKO mice are prone to autoimmune diseases and develop SS-like symptoms, such as the loss of saliva production and the infiltration of immune cells in the salivary glands as early as 4 weeks after birth [11,16]. In addition, lacrimal gland dysfunction was also observed in the SATBcKO mice after 4 weeks of age (data not shown). Although the pathophysiology is thought to vary across patients with SS, the involvement of interferon γ (IFN-γ) in SS pathogenesis has been suggested in some patients with SS and the SS mice model [19,20,21]. Therefore, we first quantified IFN-γ expression in the salivary glands of the SATB1cKO and wild type (WT) mice. The quantitative PCR analysis showed that IFN-γ expression was upregulated in the salivary glands of the SATB1cKO mice even at 3 weeks of age, a week earlier than the onset of SS, which led to the reduction in saliva production (Figure 2a). CD4^+^ helper T (Th) cells infiltrate the salivary glands of the SATB1cKO mice [16]. We examined the expression of signature cytokines produced by these Th cells. We observed that most Th cells in the salivary glands of the SATB1cKO mice were IFN-γ-producing Th1 cells (Figure 2b,c). The percentage of Th1 cells in the total CD4^+^ T cell population that infiltrated the salivary glands of the SATB1cKO mice was not significantly different but showed a minor reduction between 3 and 12 weeks after birth (Figure 2c). Distinct Th17 cells were also observed in addition to Th1 cells; however, very few Th2 cells were present in the SATB1cKO salivary glands (Figure 2b and 2c). In addition to IFN-γ, IL-6, a pro-inflammatory cytokine, was also expressed in SATB1cKO salivary glands after 4 weeks of age (Figure 2d), implying that inflammation is observed following prominent salivary gland destruction in the SATB1cKO mice.

**Figure 3:**
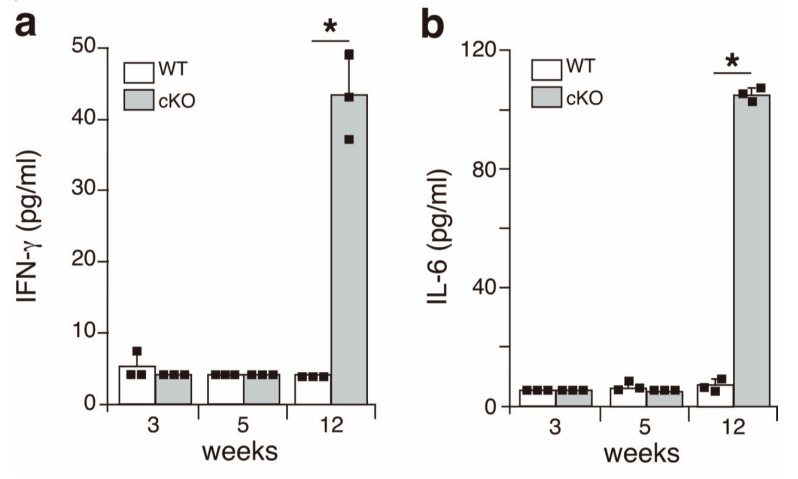
Inflammatory cytokines are present in the serum of SATB1cKO mice in the late but not the early phase of SS. The levels of IFN-γ (**a**) and IL-6 (**b**) were quantified in the serum obtained from WT (open bar) and SATB1cKO mice (filled bar) at different ages indicated in the figures. Dots in the graph indicate individual values (*n* = 3). The Student’s *t*-test was used for statistical analysis (* *p* < 0.05).

Although upregulation of the expression of IFN-γ and IL-6 was observed in the salivary glands in the early phase of SS in the SATB1cKO mice, these two cytokines were not detected at 3 weeks of age (pre-SS phase) or 5 weeks of age (early SS phase) in the serum of the SATB1cKO mice (Figure 3). In the late phase of SS, both IFN-γ (Figure 3a) and IL-6 (Figure 3b) were present in the serum of the SATB1cKO mice. Therefore, serum IFN-γ and IL-6 can be used as indicators of the late SS phase but not for the early phase of SS in the SATB1cKO mice, as is the case with anti-SSA and anti-SSB antibodies (see Figure 1).

### 2.2. l-Kynurenine Is Detected in the Serum of SATB1cKO Mice as Early as Four Weeks after Birth

**Figure 4:**
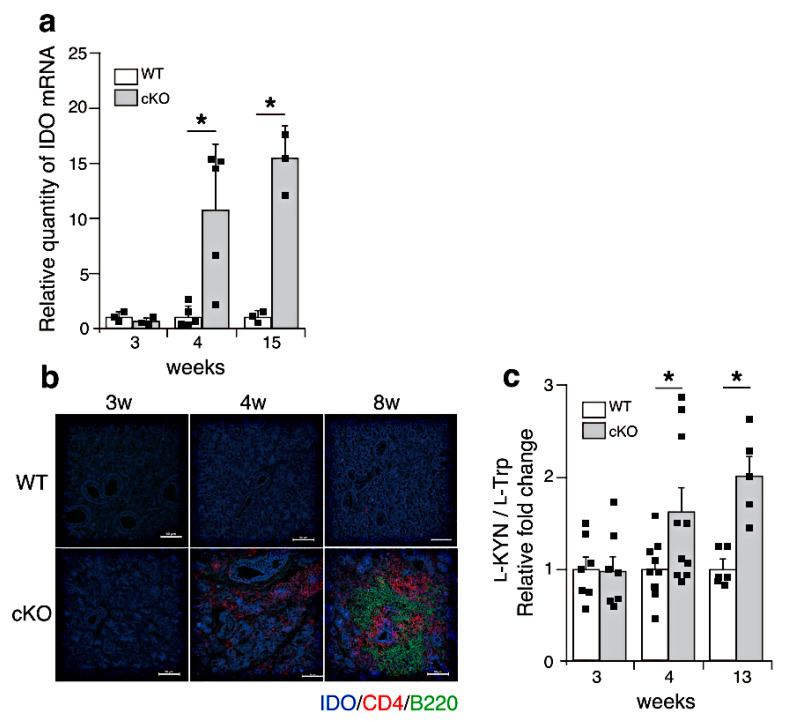
l-Trp metabolism is upregulated in SATB1cKO mice after the onset of SS. (**a**) Quantitative PCR analyses of IDO expression in the salivary glands of WT (open bar) and SATB1cKO mice (filled bar). Each result was normalized to the expression level of HPRT. Dots in the graph indicate individual values (*n* = 3–5). The Student’s *t*-test was used for statistical analysis (* *p* < 0.05). (**b**) Sections of the salivary glands from WT and SATB1cKO mice were stained with fluorescein-labeled anti-mouse IDO (blue), anti-mouse CD4 (Red) and anti-mouse B220 (green) antibodies. Scale bars represent 100 μm. (**c**) Serum was collected from WT (open bar) and SATB1cKO mice (filled bar) at different ages and analyzed for l-Trp and l-KYN levels using LC-MS/MS. The ratio of l-KYN to l-Trp in the serum was calculated. Normalized values, which are indicated as dots, in SATB1cKO mice to WT mice are shown (*n* = 6–9). The Mann–Whitney U test was used for statistical analysis (* *p* < 0.05).

Since IFN-γ demonstrates diverse effects, we hypothesized that the expression of IFN-responsive genes might be upregulated in the salivary glands of SATB1cKO mice. One of the IFN-responsive genes, IDO, was upregulated at and after 4 weeks of age in the SATB1cKO mice (Figure 4a). We next examined IDO protein expression in the salivary glands of the SATB1cKO mice. We stained sections of the salivary glands harvested from the SATB1cKO and WT mice at various ages with anti-IDO antibodies in conjunction with anti-CD4 and anti-B220 antibodies. In accordance with the results shown in Figure 4a, the IDO protein was not detected in the salivary glands of the WT mice at any age or in the 3-week-old SATB1cKO mice (Figure 4b). However, IDO expression was observed in the salivary glands of the 4- and 8-week-old SATB1cKO mice (Figure 4b). Exocrine gland dysfunction in SATB1cKO mice is initiated at 4 weeks after birth when infiltrated lymphocytes are sparsely present in the salivary gland (Figure 4b) [16]. Nevertheless, IDO expression was observed in most gland cells, not just only in the cells around the foci with infiltrated lymphocytes (Figure 4b), suggesting that IDO expression might indeed be induced by soluble factors, such as IFN-γ.

IDO is an enzyme that degrades l-Trp, an essential amino acid, into active metabolites such as l-KYN [22]. We next examined the presence of l-KYN in the serum of the WT and SATB1cKO mice using mass spectrometry. We found that l-KYN levels were increased in the serum of the SATB1cKO mice as early as 4 weeks of age (Figure 4c) when the dysfunction of salivary and lacrimal glands was observed. This finding suggests that l-KYN could be a serum biomarker for the early phase of SS.

### 2.3. IDO Expression Is Induced in SATB1cKO Mice in an IFN-γ-Dependent Manner

**Figure 5:**
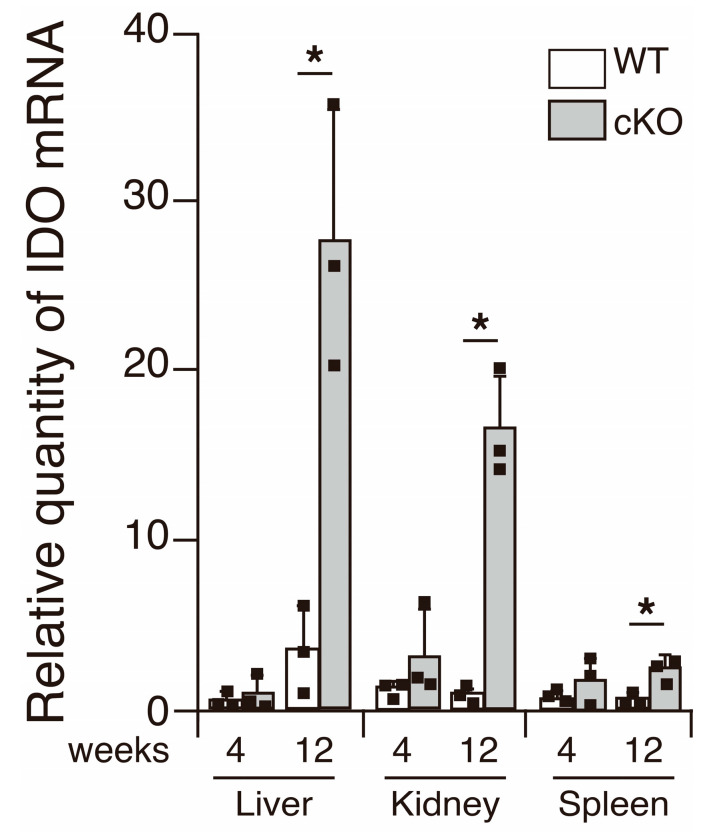
IDO expression in various organs. IDO expression was examined using quantitative PCR analyses performed with total RNA purified from the liver, kidney and spleen of WT (open bar) and SATB1cKO mice (filled bar) at 4 and 12 weeks of age. Each result was normalized to the expression level of HPRT. Dots in the graph indicate individual values (*n* = 3). The Student’s *t*-test was used for statistical analysis (* *p* < 0.05).

SATB1cKO mice develop SS and SLE. However, indicators of SLE, such as anti-dsDNA antibodies in the serum and deposition of immune complexes in the kidney, are observed after 12 weeks of age [11]. Therefore, it is plausible that IDO is expressed systemically after a certain age in SATB1cKO mice. To address this issue, we examined the IDO mRNA expression in various organs of the WT and SATB1cKO mice at 4 and 12 weeks of age, at the onset of symptoms of SS and SLE, respectively [11,16]. IDO expression was slightly increased in the spleen of the SATB1cKO mice 4 weeks after birth (Figure 5). In the 12-week-old SATB1cKO mice, a significantly higher IDO expression was observed in the liver and kidney of the SATB1cKO mice than that in the WT mice (Figure 5), implying that IDO expression may be induced in multiple organs when an autoimmune reaction activates systemic inflammatory responses. 

**Figure 6:**
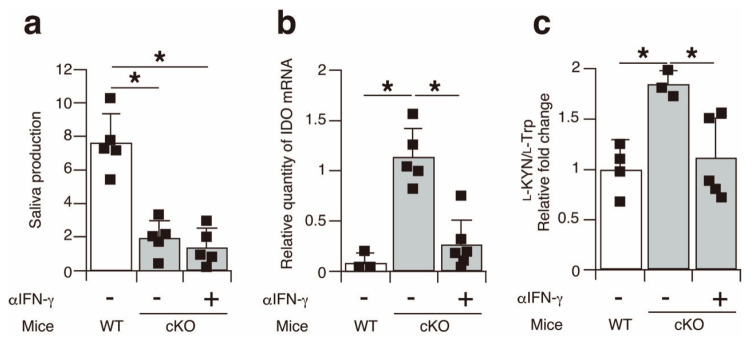
IDO expression in the salivary glands of SATB1cKO mice is upregulated in an IFN-γ-dependent manner. (**a**) Saliva production was measured in WT mice (open bar) and SATB1cKO mice (filled bar) with or without injection of anti-IFN-γ antibody. (**b**) Quantitative PCR analyses of IDO expression in the salivary glands harvested from WT mice (open bar) and SATB1cKO mice (filled bar) with or without injection of anti-IFN-γ antibodies. Each result was normalized to the expression level of HPRT. (**c**) Serum was collected from WT mice (open bar) and SATB1cKO mice (filled bar) with or without injection of anti-IFN-γ antibodies and analyzed for l-Trp and l-KYN levels using LC-MS/MS. The ratio of l-KYN to l-Trp in the serum was calculated. Normalized values in SATB1cKO mice to WT mice are shown. Dots in the graphs indicate individual values (*n* = 3–6). The Student’s *t*-test was used for statistical analysis (* *p* < 0.05).

Finally, to elucidate the causative relationship between IFN-γ and IDO expression in the salivary glands of the SATB1cKO mice, we intraperitoneally injected neutralizing IFN-γ antibodies into the 3-week-old SATB1cKO mice every 3 days, with a total of 20 injections, to inhibit IFN-γ function. No improvement in saliva production was observed in the SATB1cKO mice injected with anti-IFN-γ antibodies (Figure 6a), despite reports stating that IFN-γ is necessary for SS pathogenesis in some mouse models [21]. Nevertheless, IDO expression was significantly lower in the salivary glands of the anti-IFN-γ-treated SATB1cKO mice (Figure 6b). In addition, serum l-KYN levels decreased in the SATB1cKO mice following the anti-IFN-γ-antibody injection (Figure 6c). These results demonstrate that the induction of IDO expression in the salivary glands of the SATB1cKO mice is mediated by IFN-γ. Overall, the results of this study suggest that serum l-KYN can be a useful marker for the diagnosis of SS in the early phase before the generation of autoantibodies.

## 3. Discussion

Currently, there is no effective treatment for SS. Therefore, symptomatic treatment for dry mouth and dry eye is important for maintaining the quality of life of patients with SS [23]. Although autoimmune responses cause SS, treatment with immunosuppressive drugs has not successfully treated the disease [23]. One possible reason for this is that the exocrine function already deteriorates at the time of diagnosis, and exocrine dysfunction is irreversible. In this case, some immunosuppressive treatments, which have not been proven efficacious in previous clinical trials, might cease SS disease progression if administered early. Therefore, the identification of useful biomarkers for early SS diagnosis is a crucial first step in the development of novel treatments for SS. In this study, we demonstrated that an increase in serum l-KYN levels could be a useful marker for the diagnosis of SS.

*IDO* was identified as a type I IFN response gene that mediates the generation of l-KYN from l-Trp in the l-Trp metabolism pathway, while also demonstrating immunosuppressive functions [18,22]. Therefore, it seems that the upregulation of *IDO* in the salivary and lacrimal glands of SATB1cKO mice after the onset of SS symptoms is a negative feedback reaction against autoimmunity. IFN-γ (known as type II IFN) also mediates the induction of IDO expression in various cell types [24,25]. This expression is induced maybe because the promoter of the *IDO* gene possesses IFN-stimulated response elements (ISRE) and IFN-γ-activated sequences, which form sites for the binding of IRFs and Stats, respectively [26,27,28,29,30]. In addition, the administration of anti-IFN-γ neutralizing antibodies in the SATB1cKO mice significantly suppressed the upregulation of IDO expression in the salivary glands (Figure 6b), suggesting that the IFN-γ produced by Th1 cells might be an inducer of IDO expression in exocrine gland cells. Importantly, SS patients exhibit higher IFN-γ levels and enhanced Th1 activity in salivary glands compared to non-SS sicca patients [19,20], implying that the presence of l-KYN in the serum can be used as a marker to distinguish between autoimmune-mediated sicca (i.e., SS) and non-SS sicca. Notably, salivary gland dysfunction in the SATB1cKO mice was not improved by anti-IFN-γ antibody injections (Figure 6a). In this sense, it is worth knowing that no significant improvement in SS symptoms was observed in a phase II study examining the effects of an inhibitor of Jak1, an indispensable signaling molecule whose action is mediated via IFN-γ receptors [23]. Therefore, SS pathogenesis in SATB1cKO mice might be similar to that observed in human SS. Since lymphopenic RAG2-deficient mice develop SS after being injected with T cells derived from SATB1cKO mice [16], some T cell factors other than IFN-γ might be involved in SS pathogenesis in the SATB1cKO mice. Controversially, in another SS model of non-obese diabetic (NOD) mice, SS symptoms were not observed in the absence of IFN-γ or IFN-γ receptor genes [21]. In addition, Zhou et al. showed that the neutralization of IFN-γ improved salivary gland dysfunction in NOD mice [31]. Accordingly, the pathogenesis of SS may vary in different mouse models. 

In SATB1cKO mice, l-KYN can be detected in the serum as early as 4 weeks after birth (Figure 4c), although diagnostic factors in several SS classification criteria [32,33,34], such as anti-SSA or anti-SSB antibodies, are only detected after 8 weeks of age [16]. IDO activity in SS patients is higher than that in healthy controls; however, IDO expression in each patient was not addressed [35]. Maria et al. demonstrated that IDO expression is upregulated in the peripheral blood of some SS patients [36]. However, it is not clear whether the upregulation in IDO expression is a result of an autoimmune reaction in the exocrine glands. In addition, it has not been elucidated whether IDO is upregulated in the exocrine glands during disease development in patients with SS. Therefore, further studies are necessary to gain an insight into the clinical importance of IDO activity in patients with SS.

## 4. Materials and Methods

### 4.1. Mice

SATB1^fl/fl^Vav-Cre^+^ mice were generated as previously described [16,37]. Vav-Cre mice [38] were purchased from Jackson Laboratory (Bar Harbor, ME, USA). RAG2^-/-^ mice were maintained in the laboratory. C57BL/6 mice were obtained from CLEA Japan (Tokyo, Japan). All mice used in this study had a C57BL/6 background and were maintained under specific pathogen-free conditions at the Toho University School of Medicine animal facility. The Toho University Administrative Panel approved all experiments using mice for Animal Care (21-52-435) and recombinant DNA (21-52-440). 

### 4.2. Flow Cytometry

Antibodies used for cell surface and intracellular staining were as follows: anti-CD4-PE-Cy7 (GK1.5), anti-CD8-APC-Cy7 (53-6.7), anti CD16/32 (93), anti-IL-17-PE (TC11-18H10.1) and anti-IFN-γ-FITC (XMG1.2) were obtained from BioLegend. Anti-TCRβ-APC (H57-597) was obtained from Tombo Bioscience. Salivary glands were enzymatically digested, and infiltrating cells were isolated as previously described [16]. For intracellular staining of IL-17 and IFN-γ, the BD Cytofix/Cytoperm^TM^ Fixation/Permeabilization Kit (BD) was used according to the manufacturer’s protocol. Stained cells were analyzed using an LSRFortessa^TM^ Flow Cytometer (BD), and the data were analyzed with FlowJo software (version 9.8.1; Tree Star, Ashland, OR, USA).

### 4.3. RNA Extraction and Real-Time PCR

Total RNA was extracted using Isogen II (Nippon Gene, Tokyo, Japan), and the RNA concentration was determined spectrophotometrically at 260 nm. First-strand synthesis was performed using a High-Capacity cDNA Reverse Transcription kit (Applied Biosystems, Foster City, CA, USA). Quantitative PCR was performed using the TaqMan gene expression assay kit, following the manufacturer’s instructions (Applied Biosystems) on an ABI 7500 fast system (Applied Biosystems). The following primers were used (Applied Biosystems): Hprt, Mm00446968_ml; Ido1, Mm00492590_ml; Ifng, Mm00801778_ml; and Il6, Mm00446190_ml. Expression measurements were determined using the delta–delta Ct methods and normalized to HPRT levels. 

### 4.4. Cytokine Assay

The protein expression of inflammatory cytokines was measured using the HQPLEX Analyte Kit (Cellector, San Diego, CA, USA), according to the manufacturer’s instructions. The results were analyzed using BeadLogic software (Inivai Technologies, Mentone, Australia).

### 4.5. Histopathology and Immunohistochemistry

For microscopic immunofluorescent analysis, salivary glands were frozen in O.C.T. compound (Tissue-Tek, Sakura Finetek, Tokyo, Japan), sliced at a 6-micrometer thickness, and fixed with 4% PFA for 10 min at room temperature (RT), washed and blocked with phosphate-buffered saline (PBS) containing 5% BSA (Merck, Darmstadt, Germany) and 5% rat serum (ImmunoBioScience Corp., Mukilteo, WA, USA) for 1 h at RT, and then incubated with a rat anti-mouse IDO antibody (BioLegend, San Diego, CA, USA) at 4 °C overnight. The sections were further incubated with Alexa 647-conjugated anti-rat IgG (H+L) (Molecular Probes Eugene, OR, USA) for 1 h at RT. The sections were then stained with Alexa 594-conjugated anti-mouse CD4 (GK1.5) and Alexa 488-labeled anti-mouse B220 (RA3-6B2), purchased from BioLegend, for 2 h at 4 °C. Stained sections were mounted with Dako Fluorescent Mounting Medium (Dako, Santa Clara, CA, USA) and examined using an A1R confocal laser-scanning microscope (Nikon, Tokyo, Japan).

### 4.6. Detection of l-Trp and l-KYN in Serum Using Liquid Chromatography-Tandem Mass Spectrometry (LC-MS/MS)

Mouse serum samples for l-Trp and l-KYN measurements were prepared according to our previous report [39,40] with minor modifications. Briefly, l-Trp and l-KYN concentrations were determined using LC-MS/MS after derivatization with (R)-DBD-PyNCS. The serum ratio of l-KYN to l-Trp concentrations ([l-KYN]/[l-Trp]) was calculated and compared between SATB1cKO and WT mice. The results shown in the figures are relative [l-KYN]/[l-Trp] ratios in SATB1cKO mice against the value in WT mice.

### 4.7. In Vivo Neutralization of IFN-γ Functions

To block IFN-γ in vivo, mice were injected intraperitoneally with 200 μg of anti-mouse IFN-γ antibody (InVivoMAb, Bio X Cell, Lebanon, NH, USA) every 3 days for 60 days, as described previously [41].

### 4.8. Measurement of Saliva Volume

The mice were anesthetized using an intraperitoneal (i.p.) injection of a mixture of 0.75 mg/Kg medetomidine (Nippon Zenyaku Kogyo, Koriyama, Japan), 4 mg/kg midazolam (SANDOZ, Yamagata, Japan) and 5 mg/kg butorphanol tartrate (Meiji Seika Pharma, Tokyo, Japan). To stimulate saliva secretion, 0.5 mg/kg pilocarpine hydrochloride (Sigma-Aldrich, St. Louis, MO, USA) was intraperitoneally administered. Saliva was collected using a micropipette for 15 min. The volume of saliva was normalized to the bodyweights of the mice.

### 4.9. Statistical Analysis

Statistical analysis was performed using Student’s *t*-test to compare means, or the Mann–Whitney U test with the assumption of unequal variance and a confidence level of 95%.
